# The Law Affecting Medical Practitioners.—III

**Published:** 1902-12-06

**Authors:** J. E. R. Stephens

**Affiliations:** Barrister-at-Law, Author of "Digest of Public Health Cases"


### THE LAW AFFECTING MEDICAL PRACTITIONERS.—III.

By J. E. R. Stephens, Barrister-at-Law, Author of " Digest of Public Health Cases."

Defamation of Character.

A regular professional practitioner is entitled to recover a pecuniary compensation against any person who, without reference to his private character, has maliciously imputed to him, either by words or writing, a want of qualification, attention, skill, or capacity, or any misconduct by which he is likely to be prejudiced in the profits of his profession. No man may disparage or destroy the reputation of another. Every man has a right to have his good name maintained unimpaired. Words which produce, in any given case, appreciable injury to the reputation of another are called defamatory. Defamatory words, if false, are actionable. False defamatory words, if written and published, constitute a libel; if spoken, a slander. Words which on the face of them must injure the reputation of the person to whom they refer are clearly defamatory, and, if false, are actionable, without proof that any particular damage has followed from their use. Words, on the other hand, which merely might tend to injure the reputation of another are *prima facie* not defamatory, and even though false are not actionable, unless as a matter of fact some appreciable injury has followed from their use. No general rule can be laid down defining absolutely, and once for all, what words are defamatory and what are not. Words which would seriously injure A.'s reputation, might do B.'s no harm. Each must be decided mainly on its own facts. In each case the question will be:—Have the defendant's words appreciably injured the plaintiff's reputation? Such injury may be either presumed from the nature of the words themselves, or proved by evidence of their consequences. It will be presumed from the nature of the words themselves (*a*), if the words being written and published, or printed and published, disparage the plaintiff or tend to bring him into ridicule and contempt. (*b*) If the words being spoken, (1) charge the plaintiff with the commission of a crime (2) impute to the plaintiff a contagious disorder tending to exclude him from society; (3) are spoken of the plaintiff in the way of his profession or trade, or disparage him in an office of public trust, or (4) impute unchastity or adultery to any woman or girl. In all such cases the words are said to be actionable *per se*, because on the face of them they clearly must have injured the plaintiff's reputation. But in all other cases of spoken words the fact that the plaintiff's reputation has been injured thereby must be proved at the trial by evidence of the consequences that directly resulted from their utterances. Such evidence is called " evidence of special damage," as distinguished from that general damage which the law assumes, without express proof, to follow from the employment of words objectionable *per se*. The intention or motive with which the words were employed is, as a rule, immaterial. If the defendant has in fact injured the plaintiff's reputation he is liable, although he did not intend so to do, and had no such purpose in his mind when he spoke or wrote the words. Such proof can only go to mitigate damages. Sometimes, however, it is a man's duty to speak fully and freely, and without thought or fear of the consequences; and then the above rule does not apply. The words are privileged by reason of the occasion on which they were employed; and no action lies therefore, unless it can be proved that the defendent was actuated by some wicked or indirect motive. But in all other cases malice in fact need never be proved at the trial; the words are actionable if false and defamatory, although published accidentally or inadvertently, or with an honest belief in their truth. False defamatory words then, if spoken, constitute a slander; if written and published, a libel.

It is libellous to write of a medical man that he is a quack, or that he prepares quack medicines; but not that he has met homœopathists in consultation. It is libellous to describe a medical practitioner in print as "The Harley Street Quack, Physician Extraordinary to several ladies of distinction." The plaintiff extensively advertised a new cure for consumption. The *Pall Mall Gazette* denounced him as an impostor and quack. Chief Justice Cockburn directed the jury that if the defendant really believed that the plaintiff's system of treatment was a delusion, " then he had a right to maintain that it was so; and that, even if in drawing inferences of imposture and bad intention, he fell into error, yet if he wrote honestly, and with the intention of exercising his vocation as a public writer fairly and with reasonable moderation and judgment, he is entitled to the verdict." (*Hunter* v. *Sharp*, 15 L.T. 421.) To impute immorality or adultery to a beneficed clergyman is actionable; for it is ground of deprivation, but not so in the case of a physician. A declaration for slander alleged that defendant used words imputing adultery to plaintiff, a physician, and the words were laid to have been spoken " of him in his profession." No special damage was laid. After verdict for plaintiff, judgment was arrested, because such words, merely laid to be spoken of a physician, are not actionable without special damage: and if they were so spoken as to convey an imputation upon his conduct in his profession, the declaration ought to show how the speaker connected the imputation with the professional conduct (*Ayre* v. *Craven*, 2 A. & E. 2).

It is actionable without proof of special damage to accuse any physician, surgeon, accoucheur, midwife, or apothecary with having " caused the death of any patient through his ignorance or culpable negligence." Also to call a practising medical man "a quack-salver," or "an empiric," or a "mountebank." In *Southee* v. *Denny* (17 L.J. Ex. 151) it was held objectionable to say of a surgeon to his patient, "I wonder you had him to attend you. Do you know him? He is not an apothecary; he has not passed any examination; he is a bad character; none of the medical men here will meet him. Several persons have died that he has attended, and there have been inquests held on them." The Court were inclined to think the words, ' He is a bad character; none of the medical men here will meet him," were actionable by themselves. Also to charge any medical man or apothecary with either ignorantly or unskilfully administering the wrong medicines or medicine in excessive doses. But it is not actionable *per se* to say of a surgeon, "He poisoned the wound of his patient" without some averment that this was improper treatment of the wound; for else "it might be for the cure of it." Nor is it actionable *per se* to call a person who practises medicine without full legal qualification "a quack," or "an impostor;" for the law only protects lawful employments. Nor to charge a physician or surgeon generally with "malpractice"; not stating that he caused his patient's death by malpractice. To say of an "accoucheuse," "A lady who has established a medical college at —— has issued a prospectus, in which my name appears as president. I have sanctioned the issue of no prospectus with my name in it. I wish to know what remedy I have," was held no slander on her in the way of her trade. The plaintiff was a surgeon and accoucheur; the defendant told one of his patients that the plaintiff's female servant had had a child by him; and the patient consequently ceased to employ the plaintiff. It was held that, special damages being proved, the action lay.

The truth of any defamatory words is, if pleaded, a complete defence to any action of libel or slander (though alone it is not a defence in a criminal trial). The onus, however, of proving the truth of the words lies on the defendant. The falsehood of all defamatory words is presumed in the plaintiff's favour, and he need give no evidence to show they are false; but the defendant can rebut this presumption by giving evidence in support of his plea. If the jury are satisfied that the words are true in substance and in fact they must find for the defendant, though they feel sure that he spoke the words spitefully and maliciously. On the other hand, if the words are false, and there be no other defence, the jury must find for the plaintiff, although they are satisfied that the defendant *bona fide* and reasonably believed the words to be true at the time he uttered them. But the whole libel must be proved true; it will be no defence to the action to prove that a part merely is true. But where the gist of the libel consists of one specific charge which is proved to be true, the defendant need not justify every expression which he has used in commenting on the plaintiff's conduct. When the libel complained of exposed the "homicidal tricks of those ignorant and impudent scamps who had the audacity to pretend to cure all diseases with one kind of pill," asserted that "several of the rotgut rascals had been convicted of manslaughter, and fined and imprisoned for killing people with enormous doses of their universal vegetable boluses," and characterised the plaintiffs' system as "one of wholesale poisoning;" and it was proved at the trial "that the plaintiffs' pills, when taken in large doses, as recommended by the plaintiffs, were highly dangerous, deadly and poisonous," and "that two persons had died in consequence of taking large quantities of them; and that the people who administered these pills were tried, convicted, and imprisoned, for the manslaughter of these two persons;" this was held a sufficient justification, although the expressions, "scamps," "rascals," and "wholesale poisoning," had not been fully substantiated; the main charge and gist of the libel being amply sustained.

As we have before stated, in certain cases, even though the the matter complained of is defamatory, in the interests of public policy no liability attaches to the publication thereof—in other words, the occasion is privileged. The question whether the occasion is privileged, if the facts are not in dispute, is a question of law only, for the judge, not for the jury. If there are questions of fact in dispute upon which this question depends, they must be left to the jury, but when the jury have found the facts, it is for the judge to say whether they constitute a privileged occasion. A confusion is often made between a privileged communication and a privileged occasion. It is for the jury to say whether a communication was privileged; but the question whether an occasion was privileged is for the judge, and that question only arises when there has been publication to a third party. In other words, "whether or not the occasion gives that privilege is a question of law for the judges; but whether or not the party has fairly and properly conducted himself in the exercise of it (*i.e.*, whether he has acted maliciously), is a question for the jury." If the judge rules that the occasion is privileged, and if there is no evidence of malice to go to the jury, it is his duty to enter judgment for the defendant. On the other hand, if the occasion is not privileged, or if there is any evidence of malice, then the case must be left to the jury. It is, however, frequently a matter of great difficulty to determine whether or not a particular occasion is privileged. As was pointed out by Chief Justice Erle in *Whiteley* v. *Adams* (15 C.B N.S. 418): "Judges who have had from time to time to deal with questions as to whether the occasion justified the speaking or the writing of defamatory matter, have all felt great difficulty in defining what sort of social or moral duty, or what amount of interest, will afford a justification; but all are clear that it is a question for the judge to decide." Again, in the words of Lord Justice Lindley in *Stuart* v. *Bell* (1891, 2 Q.B. 346): "The reason for holding any occasion privileged is common convenience and welfare of society, and it is obvious that no definite lines can be drawn so as to mark off with precision those occasions which are privileged, and separate them from those which are not."

